# Using Two Different Approaches to Assess Dietary Patterns: Hypothesis-Driven and Data-Driven Analysis

**DOI:** 10.3390/nu8100593

**Published:** 2016-09-23

**Authors:** Ágatha Nogueira Previdelli, Samantha Caesar de Andrade, Regina Mara Fisberg, Dirce Maria Marchioni

**Affiliations:** Department of Nutrition, School of Public Health, University of São Paulo-USP, São Paulo 01246-904, Brazil; samantha.caesar@gmail.com (S.C.d.A.); rfisberg@usp.br (R.M.F.); marchioni@usp.br (D.M.M.)

**Keywords:** adolescents, dietary approaches, dietary patterns, Brazilian Healthy Eating Index Revised (BHEI-R), factor analysis, diet quality

## Abstract

The use of dietary patterns to assess dietary intake has become increasingly common in nutritional epidemiology studies due to the complexity and multidimensionality of the diet. Currently, two main approaches have been widely used to assess dietary patterns: data-driven and hypothesis-driven analysis. Since the methods explore different angles of dietary intake, using both approaches simultaneously might yield complementary and useful information; thus, we aimed to use both approaches to gain knowledge of adolescents’ dietary patterns. Food intake from a cross-sectional survey with 295 adolescents was assessed by 24 h dietary recall (24HR). In hypothesis-driven analysis, based on the American National Cancer Institute method, the usual intake of Brazilian Healthy Eating Index Revised components were estimated. In the data-driven approach, the usual intake of foods/food groups was estimated by the Multiple Source Method. In the results, hypothesis-driven analysis showed low scores for Whole grains, Total vegetables, Total fruit and Whole fruits), while, in data-driven analysis, fruits and whole grains were not presented in any pattern. High intakes of sodium, fats and sugars were observed in hypothesis-driven analysis with low total scores for Sodium, Saturated fat and SoFAA (calories from solid fat, alcohol and added sugar) components in agreement, while the data-driven approach showed the intake of several foods/food groups rich in these nutrients, such as butter/margarine, cookies, chocolate powder, whole milk, cheese, processed meat/cold cuts and candies. In this study, using both approaches at the same time provided consistent and complementary information with regard to assessing the overall dietary habits that will be important in order to drive public health programs, and improve their efficiency to monitor and evaluate the dietary patterns of populations.

## 1. Introduction

The use of dietary patterns to assess dietary intake has become increasingly common in nutritional epidemiology studies due to the complexity and multidimensionality of the diet [1,2,3]. Currently, two major approaches have been widely used: hypothesis-oriented and data-driven analysis. Both have different purposes that are useful for deriving meaningful dietary patterns that can be associated or not to a particular health outcome. The first one, also called “a priori” analysis, is based on previous information (food guides and nutritional recommendations) used to stratify a dietary pattern. Dietary indices are the most common hypothesis-oriented approaches that evaluate the adherence of population intake to nutritional recommendations. The data-driven approach, on the other hand, provides insight into the dietary behavior of participants, since the evaluation of overall dietary pattern is based on the actual population food intake. Data-driven approaches are considered important from a public health point of view because they can be used to assist with the development of food-based guidelines [2]. A common technique used is the principal components analysis (PCA), followed by factor analysis. The PCA aims to reduce a large set of correlated variables into a small set of non-correlated variables that contains the same information of the larger one [4], revealing the underlying structure within diets of the population [5]. Since the methods explore different angles of dietary intake, using both approaches simultaneously might yield complementary and useful information.

In order to establish the actual association between diet, chronic diseases and associated factors, it is essential to estimate the usual dietary intake [6,7]. It has been recognized that use of a single short-term dietary recall, such as the 24 h dietary recall (24HR), is not enough to predict the usual intake [6]. Therefore, over the past few years, several statistical methods have been developed to remove the intra-individual variability across consumption days in order to provide the usual dietary intake at population and individual levels [6,7].

Based on the usual intake, this study aimed to assess the dietary pattern using two approaches: hypothesis-oriented (assessed by the distribution of dietary index components’ scores) and data-driven analysis (assessed by exploratory principal component factor analysis). Measures of dietary patterns may be used to investigate interactions with other health behaviors to examine the determinants of eating patterns. Knowledge gathered from both approaches might allow for better targeting and intervention designs, programs, and public policies that aim to improve dietary intake of adolescents.

## 2. Methods

### 2.1. Design and Study Population

This was a cross-sectional study designed with adolescents of both genders, aged 12 to 19 years, who provided complete and reliable data from 24 h dietary recall (24HR). Adolescents were recruited from a sample drawn from the Health Survey of São Paulo (ISA-Capital) conducted in 2007. The ISA-Capital was devised to collect data on health status and access to health services, in addition to life habits, socio-economic levels and dietary conditions in a representative sample from residents of the city of São Paulo, southeastern Brazil.

Briefly, the sampling process entailed two stages: primary sampling units were census sectors, while secondary units were domiciles. Sampling selection was performed by grouping the sectors into three sub strata according to percentage of heads of households with university-level education: less than 5%, 5% to 24.9%, and 25% or greater. The interviews were planned according to eight domains composed of age groups and gender of individuals: less than 1 year of age (one domain), 1 to 11 years of age (another domain), 12 to 19 years of age (one domain for each gender), 20 to 59 years of age (one domain for each gender) and 60 years of age and over (one domain for each gender). Initially, 813 adolescents were interviewed and those within the age bracket (younger than 20 years) at the start of data collection for the present study (March/2007) were invited to participate. This gave a sample of 412 adolescents. From that, 2.7% (*n* = 11) refused to take part, 15.3% (*n* = 63) had changed their address and could not be located, 15.7% (*n* = 65) were not home during three separate visits at different times and days of the week, 2.2% (*n* = 9) did not provide all the socio-economic information and *n* = 6 did not provide consistent 24HR information. Thus, for the “a priori” analysis, the final sample included 267 adolescents, 136 males and 131 females. As 2.7% (*n* = 12) did not provide the Food Frequency Questionnaire (FFQ) information, “a posteriori” analysis comprised 229 adolescents, 117 males and 112 females, with no differences between the sample of both analyses (*p* > 0.05). The proportion of individuals in the three strata did not differ between initial and final study samples (*p* = 0.19).

### 2.2. Data Collection

Interviews were conducted between 2007 and 2008. Information on demographic, socioeconomic, and lifestyle factors were obtained through structured standard questionnaires administered by trained interviewers. Dietary intake was assessed by 24 h dietary recall method (24HR) applied according to the multiple-pass method [8]. The first 24HR and the questionnaire information were collected at adolescents’ households. The following 24HR data collection were carried out by telephone; thus, the participants provided a minimum of one and a maximum of five 24HR. To ensure the quality of dietary intake information, at the end of the interview, data were critically reviewed to identify any failures relating to descriptions of foods or preparations consumed and their apportioning and quantification. If necessary, the interviewer asked these questions and completed the information. Data were analyzed using Nutrition Data System for Research (database version, 2007, NCC, University of Minnesota, Minnesota, MN, USA). After that, energy and macronutrient values of foods and ingredients were checked. All 24HR in which the estimation of total energy intake was higher than the 95th percentile or lower than the 5th percentile were checked just to ensure data quality and that no one was excluded.

Data from a validated Food Frequency Questionnaire (FFQ), carried out by telephone, were used. Briefly, the FFQ was elaborated based on data of consumption from a previous release of the Healthy Survey-São Paulo carried out in 2003, and it was prepared through a statistical analysis approach proposed by Block et al. [9,10]. The FFQ comprised 67 food items in order to capture the frequency of consumption of foods and food groups. The information from the FFQ was used in two parts of the data-driven analysis: (1) to determine the food groups used in the factor analysis and (2) as covariate information to improve the modeling of consumption probability and frequency, which is described in the data analysis section.

The study was approved by the Human Research Ethics Committee of the School of Public Health at the University of São Paulo (process No. 1959, on 8 July 2009). In addition, the parents or guardians signed a consent form to approve the adolescent participation.

### 2.3. Diet Quality Assessment

Two approaches were used to assess the dietary patterns: hypothesis-oriented and data-driven analyses.

#### Hypothesis-Oriented Analysis

The hypothesis-oriented analysis, based on a validated Dietary Index called the Brazilian Healthy Eating Index–Revised (BHEI-R), was used to assess the overall dietary quality [11,12]. The BHEI-R was developed to (1) capture the overall diet quality; (2) measure dietary risk factors for chronic diseases; (3) evaluate the dietary intake of individuals and/or population and (4) monitor the population intake [11].

The BHEI-R score is the sum of individual scores for 12 components in which nine food groups were based on Dietary Guidelines for Brazilians [13]. The other components were based on recommendations from the World Health Organization (WHO), the Institute of Medicine (IOM), the Healthy Eating Index 2005 (HEI-2005) and the Brazilian Cardiology Society [11]. The components were expressed on intake per 1000 calories or per calorie basis in order to represent the diet quality rather than the diet quantity [11,13].

For all components, intakes at a level of the standard or higher are assigned the maximum number of total points allotted (5, 10 or 20 points) and intermediate values represent the proportion of the component in the intake. A total of nine components were based on the food group recommendations of the Dietary Guidelines for Brazilians, with a maximum score of 5 points (Total Grains; Whole Grains; Dark-Green and Orange Vegetables and Beans; Total Vegetables; Total Fruit; and Whole Fruit) or 10 points (Dairy Products; Meat and Beans; and Oil, which included monounsaturated and polyunsaturated fat, nuts and fish oils). A total of 10 points were allowed for the components Saturated Fat and Sodium, based on nutrient intake. At last, 20 points are granted for the SoFAAS (calories from solid fat, alcohol and added sugar) component that comprises the total calories from solid fats, alcoholic beverages and added sugars. Accordingly, the total BHEI-R scores ranges from 0 to 100.

### 2.4. Data-Driven Analysis

The data-driven approach was based on factor analysis with principal component factor extraction. For this, all foods, drinks and recipes consumed by the adolescents in the 24HR were entered into the Nutrition Data System for Research (NDS-R, version 2007), which provided the energy contribution of all the foods, drinks, and ingredients consumed by each adolescent. Foods consumed were located in one of the 67 food items of the FFQ [10]. In the next step, the remaining foods and the food groups were combined according to nutritional similarity and also to represent particular dietary habits of the São Paulo population. A small number of food groups, consumed by less than 5% of the sample population, were excluded from the analysis: alcoholic beverages, bouillons, popcorns, nuts, jams, soymilk, soups, potatoes and mixed dishes (salt pies, risottos, pates and others). Previously, the factor analysis was tested on the 44 groups. Nevertheless, to improve the analysis, some groups were combined, totaling 30 items defined as: beans and legumes; rice; sugar; coffee and tea; pastry and sandwiches; butter and margarine; beef; cookies; whole milk; breads; natural flavorings; chocolate powder; vegetables; cheeses; salad dressing; candies; white meat; processed meats and cold cuts; sodas; semi-skimmed and skimmed milk; juices; pasta; sauces; eggs; fruits; ice creams; cakes and pies; snacks; pork meats; yogurt and similar (Table 1).

### 2.5. Statistical Analysis

#### Hypothesis-Oriented Analysis

In the hypothesis-oriented approach, the method proposed by the American National Cancer Institute (NCI) was used to estimate the population distribution of each BHEI-R component score [14]. This method was chosen because of its capability for estimating usual dietary intake of nutrients and foods, including episodically consumed foods by the populations. Briefly, the NCI method is based on a joint bivariate model for nutrient/food group (numerator) and energy (denominator) [6,14]. In this ratio, both numerator and denominator were worked in terms of kilocalories, with exception to the sodium that was worked in mg. Then, the distribution of the ratio of usual intakes was applied to estimate the distribution for the components scores [14]. The NCI method requires at least two days of short-term dietary measurements on a random subsample of the target population to produce the usual intake estimates. Further information about the NCI method was published elsewhere [14]. For the models, we investigated the association between the BHEI-R components scores and each one of the following variables: age (years); gender (male or female), marital status of the adolescents and marital status of the head of the family (living alone or living with a partner/married); smoking habits (smoker, former smoker or non-smoker); alcoholic beverages frequency (less than one day a month, 1 to 2 days a month or more than one day a week); and family head’s schooling (low: 0 to 8 years, medium: 8 to 11 years or high: more than 11 years). Adolescents’ nutritional status was classified based on Body Mass Index (BMI) cut-off points, according to gender and age proposed by Cole et al. [15]. Anthropometric measurements of self-reported weight and height were used to calculate BMI. According to their weight status, the adolescents were classified as “underweight”, “normal weight”, “overweight” or “obese”. Per monthly capita household income was defined in three categories: (low: less than a minimum wage, medium: 1 to 2 minimum wages or high: more than 2 minimum wages), according to the value of the minimum wage at the period of collection (about US$75.00).

In the first step of the analyses, all socio-demographic variables were evaluated, identifying that gender and family head’s schooling (*p* < 0.05) were associated with the BHEI-R components scores, so the estimation of the components scores distribution was adjusted by these co-variables. Because the data of the present study was not weighted, it was not possible to calculate the standard error of the mean BHEI-R score [16]. The results were stratified by gender.

A probability value of 0.05 was considered statistically significant. Values in the text were expressed as mean and standard deviation (SD) or estimated as percentiles. The SAS code for performing the analysis is available at [17].

### 2.6. Data-Driven Analysis

The web-based statistical modeling technique Multiple Source Method (MSM), proposed by the European Prospective Investigation into Cancer and Nutrition (EPIC), was used to estimate the adolescents’ usual intake. This method was chosen because of its capability for estimating usual dietary intake of nutrients, foods and food groups by removing the within-person variance intake. Furthermore, this method is able to estimate the usual intake not only at the population but also at the individual level [7].

Briefly, the MSM method requires at least two days of short-term dietary measurements (such as 24HR) on a random subsample of the target population to produce the usual intake estimates. To apply the MSM method, the consumption of foods/food groups was assessed in terms of kilocalories (kcal). For each adolescent, the usual intake of each food group (kcal/person) was estimated that then was used to assess the dietary patterns. In addition, the FFQ data is optional in the MSM method, and it was used as covariate information to improve the estimation of consumption probability, identifying the habitual consumers and non-consumers [7]. In this step, none of the sociodemographics, anthropometric, or lifestyle characteristics were used as adjusted co-variables because they were used later in multiple linear regression.

Dietary patterns were generated by factor analysis using the principal components method to reduce the 30 food group intakes into a smaller number of underlying factors (dietary patterns) that could explain variations on dietary intake [18].

The varimax orthogonal rotation, in which the axles are maintained in 90°, was performed to simplify factor matrix and to make the data interpretation easier, generating non-related factors that can be used in later multiple analyses [19,20].

To identify the number of factors (patterns) to be retained, the eigenvalues >1.0 criterion in the first step was used. This procedure identified twelve patterns; thus, the break-point identified in the scree plot and the interpretability of the dietary patterns clearly identified two major dietary patterns.

Rotated factor loadings with an absolute value of 0.30 or more were considered as significantly contributing to each dietary pattern, in which higher loadings indicated stronger association between the food item and the dietary pattern. Then, factor scores were calculated for each adolescent, representing the level of adherence to each specific pattern.

The sample adequacy was verified by the Kaiser–Meyer–Olklin (KMO) test and by Bartlett’s sphericity test. KMO values above 0.50 and *p*-values of Bartlett’s sphericity test less than 0.05 [21,22] were considered acceptable. Dietary patterns were named according to the nature of the food groups loading highest for each of the factors.

Multiple linear regression analyses were used to determine significant associations between the identified dietary patterns and each one of the following variables: age (years); gender (male or female), marital status of the head of the family (living alone or living with a partner/married); smoking habits (smoker/former smoker or non-smoker); alcoholic beverages intake (yes or no); family head’s schooling (low–primary school, medium–secondary school, or high–complete high school); and remunerated activity by the head of the family (yes or no). Weight status was based on the cut-off points for Body Mass Index (BMI) according to gender and age proposed by [15]. Anthropometric measurements of self-reported weight and height were used to calculate BMI. According to their weight status, the adolescents were classified as “underweight”, “normal weight”, “overweight” or “obese”. Monthly per capita household income was defined in two categories: (until a minimum wage or more than 1 minimum wage), according to the value of the minimum wage at the period of collection (about US $75.00). Independent variables with *p*-values ≤ 0.20 in univariate analysis were selected for multiple regression analyses and included in the regression model by stepwise forward procedure. Variables that remained significant in the multiple regression model (*p* < 0.05) were maintained.

In the final models of the two dietary patterns, the heteroscedasticity was confirmed by the Breusch–Pagan/Cook–Weisberg test [23]. The variance inflation factors (VIF) were examined post-regression to ensure the absence of multicollinearity among independent variables in the models [24]. A maximum VIF of ten confirmed the absence of multicollinearity [25].

All statistical analyses were performed using Stata software (version 10.0, StataCorp LP, College Station, TX, USA). A probability value of 0.05 was considered statistically significant. Values in the text were expressed as mean and standard deviation (SD) or in absolute number.

## 3. Results

The mean age of target group was 17.7 years old and the standard deviation (SD) was 1.2. The sample studied was primarily normal weight (68.0%), former or non-smoker (86.9%), head of the family with remunerated activity (84.3%), 70.7% of the heads of the families lived with a partner or were married; and 64.8% received more than a minimum wage. There were similar proportions of boys (51%) and girls (49%).

Table 2 shows the estimate of each index component score distribution according to gender and for the overall population. The estimated mean BHEI-R score of the adolescents was 47.13 points. The 5th and 95th percentiles were 26.79 and 67.43 points, respectively. The overall dietary quality was similar between the genders (47.97 points for females and 46.30 for males), out of possible scores of 100 points.

Among the components whose total scores ranged from zero to five, higher mean scores were found for Total grains (4.98); and Dark green and Orange vegetables and Legumes (4.24). Lower mean scores were found for Whole grains (0.29) and Total vegetables (1.01) (Table 2).

For the components that showed total scores ranging from zero to ten, the population study tended to have higher mean scores for Meat, egg and beans (8.01 ± 1.03) and Oils (9.98 ± 0.18). The only components that were higher for the male adolescents than the females were Meat (8.16 ± 0.94 vs. 7.85 ± 1.08) and Saturated fat (6.39 ± 1.56 vs. 5.20 ± 1.86), in which the 5th percentile varied substantially between the genders (3.58 for male and 1.90 for female). A lower mean score was found for sodium (2.18 ± 1.17 points), indicating higher intake.

The SoFAAS component, that comprises total calories from solid fat, alcohol intake and added sugar, has an important role in the total BHEI-R score, since its maximum score is twice that of the other BHEI-R component and comprises up to 20% of the total index score. SoFAAS scores were similar between the genders (mean ± SD: 4.59 ± 2.87 for female vs. 4.42 ± 2.77 for male). These results potentially indicate that the adolescents’ dietary pattern was characterized by high intake of solid fats, alcoholic beverages and added sugar.

In the factor analysis, two major dietary patterns were retained, explaining 17.1% of total variance of intake. The factor loadings were presented in Table 3. The KMO was 0.51, indicating appropriateness of the factor analysis.

The first pattern explained 9.0% of the variation in food intake, which was characterized as “Traditional diet,” since it showed higher factor loading values in beans/legumes, rice, coffee/tea, sugar, butter/margarine, beef and cookies. Multiple regression models were inversely associated with the male gender (β = −0.70; *p* < 0.01), higher family head schooling (β = −0.59; *p* = 0.05) and higher family income (β = −0.34; *p* = 0.04). The final model was also adjusted for age and weight status (Table 3 and Table 4).

The second factor explained 8.1% of the variation in food intake, and it was called “Dual diet”. Higher factor loading values were found for chocolate powder, vegetables, whole milk, salad dressing, cheeses, processed meats/cold cuts, breads/toasts/crackers, candies and juices. In the multiple regression model, which was adjusted for age, higher family head schooling (β = 0.56; *p* = 0.03) and higher family income (β = 0.48; *p* = 0.01) were positively associated with “Dual diet” (Table 3 and Table 4).

There was no relationship between adolescent smoking habit, alcoholic beverage intake and adolescent’s remunerated activity and the dietary patterns identified.

In both dietary patterns, the maximum variance inflation factor was 1.12. The *p* values for the Breusch-Pagan test were *p* = 0.698 (pattern 1) and *p* = 0.503 (pattern 2).

## 4. Discussion

The purpose of the present study was to use two different approaches to investigate the dietary patterns, both of them based on usual intake of the population with the purpose of obtaining complementary information, which is useful for better understanding eating behaviors. As presented by the results, the hypothesis-driven approach showed how the population’s diet study was close to the recommendations, while the data-driven approach, taking the advantage of the complex combinations of dietary intake, showed how the adolescents were combining food items into two main patterns.

In the present study, two patterns were retained, called “Traditional diet” and “Dual diet”, which explained 9.0% and 8.1% of the variation in food intake, respectively. These percentages were similar to those found among Australian adolescents and lower in other Brazilian studies [1,2,26,27,28,29]. However, these differences are expected, since this percentage of the explained variation in food intake can be affected by the sample size, number of food items and the number of retained dietary patterns, which are arbitrary decisions [22]. These first patterns had positive loadings for foods/food groups often consumed by Brazilians at lunch and dinner (rice, bean and beef) and breakfast (coffee/tea, sugar and butter/margarine) [26,27]. Similarities between adolescents living in São Paulo and the overall Southeast Brazilian population were found for the food items that comprised the “Traditional” and “Rice and Bean” patterns, as both diets were inversely correlated with higher education level of heads of the families and higher family income [26]. Furthermore, the “Traditional diet” of adolescents living in Cuiaba (a city in the North of Brazil) was also inversely associated to higher socioeconomic levels [27]. Six other studies, conducted in the adolescent population worldwide, have evaluated the factors associated with dietary patterns, assessed by Food Frequency Questionnaires [2,27,28,29,30]. Three of them showed that healthy patterns, represented by the “Traditional” diet, were associated with higher socioeconomic status and greater maternal education, which was different from the results obtained in the present study [28,29,30]. The other two studies found no relationship between the socioeconomic indicators and the identified dietary patterns [2,31]. Furthermore, it was observed that not only the dietary patterns varied across the countries but also the determinants of a healthy diet.

The “Dual diet”, characterized by the consumption of healthy and unhealthy foods (vegetables, legumes/nuts, tea, rye, cheese/dairy product and alcoholic beverages), was positively correlated to higher family income and higher education level of the head of the family. Studies suggest that higher family budget is associated with higher intake of fat and calories, represented in this pattern by the whole milk, salad dressing, cheeses, processed meats/cold cuts and candies [32,33]. It should be noted that diets that comprise healthy and unhealthy foods, called in the present study “Dual diet”, are present in many studies that used factor analysis approaches to identify dietary patterns [1,26,34]. This could be explained by the fact that, in this approach, the names of the factors extracted are determined by the researchers, and also because different populations have different food habits, which may make it difficult to reproduce the same dietary patterns in other populations [1].

In the hypothesis-oriented approach, the Brazilian Healthy Eating Index–Revised (BHEI-R) showed poor overall diet quality, reflected by the low adherence to several recommendations of the Brazilian Dietary Guidelines. The estimated mean BHEI-R score was 47 points, which means that only 50% of the maximum score allowed was achieved. It was similar between genders (46.3 points for males and 48.0 for females). This score was lower than those reported in similar population studies conducted among adolescents in both Brazilian and American populations, 59.7 (SD = 0.4) and 62.8 (SD = 11.8), respectively [35,36].

Since the total BHEI-R score is obtained from the summary of 12 components, the evaluation of the dietary quality can also be done in order to analyze how each component behaved within their set point limits. This process is able to check how each BHEI-R component is in compliance with the various key aspects of the diet specified in Dietary Guidelines for Brazilians [13], which provides recommendations for nutrients, foods and food group intake. Thus, the dietary patterns, assessed by the factor analysis and data-driven approach, were also analyzed based on the food items that comprised each one. This analysis is useful for driving future public nutrition programs.

Based on the BHEI-R, the overall score for the Meat, egg and beans component was high (8.01 points). According to the results found by the factor analysis, it was possible to understand the eating habit of the adolescents and to identify the food source of this component: the diets consisted of Cow beef and Beans/legumes (“Traditional Diet”) and also processed meats and Cold cuts (“Dual Diet”). This scenario reflects the dietary patterns of the overall Brazilian population and for adolescents living in São Paulo that have been characterized by higher consumption of meat since the last decade [33,36].

Another important finding was the low mean score for the Sodium component (2.18 of a possible 10.0), reflecting a high intake of this nutrient among the adolescents. The high sodium intake found is in agreement with the high sodium consumption observed in the overall Brazilian population [33,37,38]. Our hypothesis is that a higher intake of industrialized products rich in sodium, such as condiments, snacks and processed meats, may explain the excessive sodium intake observed in the Brazilian population. Again, the results from data-driven analysis showed which items present in the “Traditional” and “Dual diet” could be a source of sodium, such as Butter/margarine, Cheeses, Salad dressing, Processed meats and Cold cuts [33]. This high intake of sodium is alarming, since the literature has been showing that the association between high sodium intakes is positively associated with adiposity and inflammation [39].

For the overall population, we observed a low estimated mean BHEI-R score for SoFAAS (component that comprises the total calories from solid fats, alcoholic beverages and added sugars); Total fruits; Whole fruit; Total vegetables; and Whole grains. The consumption of Total grains and Meat was higher for the overall population. Based on the dietary patterns, it was possible to identify a high intake of Butter/margarine, Sugar, Cookies, Breads/toasts and crackers, present in the “Traditional Diet”, and Chocolate powder, Whole milk, Salad dressing, Cheeses, Processed meats/cold cuts, Candies and industrialized juices, present in the “Dual Diet” can explain the low total score for SoFAAS. Moreover, in both patterns, no fruits were present, reflecting the low score for BHEI components that take these food groups into account. Despite Brazil’s great biodiversity, a low intake of vegetables was observed, since, for the factor analysis, all reported vegetables had to be grouped into one item that was present only in the “Dual Diet”. These results are in agreement with those found in the literature, which suggest an over-consumption of cookies, candies, snacks and soft drinks, increasing the intake of sugars and saturated fats by adolescents and lower intake of fruits and vegetables, resulting in higher in energy density diets, lower in fiber [36,40,41,42,43,44]. In addition, a multicentric study showed the association between low whole grain intake with worse dietary quality and their consequence on the nutritional status among adolescents from the HELENA study [45].

Some limitations and strengths should be highlighted about the results and the methodology of both approaches. In the hypothesis-oriented analysis, the estimated 5th and 95th percentiles BHEI-R scores for the Oil component (that comprises calories from vegetables oils and fat present in nuts and fish) were 10.00 points. However, it is important to elucidate that these higher scores can be explained by the fact that this component takes into account the oil used to deep fry foods, such as French fries, breaded snacks and whipped toppings [40,43]. The Oil component was developed to capture only the good source of oils, but it is not able to compute overconsumption. In this sense, the capacity of this component contributing to assess the overall dietary quality might be questionable, and further studies are needed to assess this subject in future revisions of this index. Secondly, a total of 50% of the population study had reached the maximum score (5.00) for Dark green and Orange vegetables and Beans for both genders, meaning that this component was not so accurate in capturing the differences within the population study. A probable reason for this is that the minimum amount intake necessary to obtain the maximum score (five points) is only half a portion of these food groups, which represents a consumption of only 7.5 kcal per 1000 kcal intake [11]. On the other hand, it is important to highlight that density standards (applied in the index) allow a common standard to be used, and also have the advantage of being independent of an individual’s energy and nutrient requirement. Furthermore, the density approach to setting standards allows the quality assessment of the mix of foods consumed, rather than the absolute amounts of foods consumed, which was the purpose of our manuscript.

In relation to the results from the data-driven analysis, it is important to emphasize that factor analysis has an arbitrary nature. Subjective decisions and judgments must be taken in several steps of the analysis, including the classifications of food into food groups, numbers of food groups, numbers of factors extracted, rotation methods used, and the names of the factors extracted [1]. These decisions can affect the dietary patterns composition and their association with socioeconomic, anthropometric and lifestyle characteristics. Thereby, during the factor analysis, the present study adopted similar criteria used by other researchers, allowing comparisons between the dietary patterns and their associated variables with the results of other dietary patterns studies [1,26,27]. In addition, dietary patterns derived from factor analysis generally tend to account for only a small amount of the total variance, especially when food consumption was assessed by food record (not by a Food Frequency Questionnaire-FFQ). In most of the studies, the food frequency questionnaire has been used to assess dietary patterns due to capability to evaluate the usual intake. However, in order to capture the greatest possible variety in foods consumed, we decided to apply the 24 h dietary recalls (R24h). To capture the usual intake based on the R24h, it was necessary to remove the intra-individual variability across the consumption days. This is a crucial processing step that must be applied in epidemiologic studies that aim to evaluate the dietary intake at both individual and population levels [7,14]. In this study, a repeated measure of the R24h and the FFQ data provided information on the amount of consumption and the probability of consumption, used by the MSM software (version 1.0.1, Potsdam-Rehbruecke, Nuthetal, Germany) to predict the usual dietary intake of each adolescent. To our knowledge, this is the first study in which the dietary patterns, assessed by factor analysis, were based on the usual dietary intake of a population.

## 5. Conclusions

The present findings showed that results from both approaches answered different research questions that together can be used by health professionals to improve the dietary quality of habitual eating patterns in the overall population.

Hypothesis-driven analysis observed low scores for the components Whole grains and Fruits (total and whole), while dietary patterns derived from data-driven analysis showed no consumption of fruits and whole grains. High intakes of sodium, fats and sugars were observed in hypothesis-driven analysis by low total scores for Sodium, Saturated fat and SoFAASA components. Data-driven analysis results showed the intake of several foods/food groups rich in these nutrients, such as Butter/margarine, Cookies, Chocolate powder, Whole milk, Cheese, Processed meat/cold cuts and Candies. It should be highlighted that dietary patterns derived from data-driven analysis were characterized by the consumption of important foods, such as milk and vegetables, that are essential in promoting healthy adolescent development. However, based on the hypothesis-driven results, we concluded that the majority of the adolescents did not meet the dietary guideline recommendations as reflected by the low total score of “milk” and “total vegetables” components.

Using both approaches at the same time provided consistent and complementary information with regard to assessing the overall dietary habits that will be important in order to drive public health programs, and improve their efficiency to monitor and evaluate the dietary patterns of populations.

## Figures and Tables

**Table 1:** Foods and food groups included in factor analysis. São Paulo.

Foods or Food Groups	Main Food Items
Beans and legumes	brown and black bean, chickpea, green pea, snow pea, lentil, soybean
Rice	white rice
Sugar	white sugar
Coffee, teas	coffee, cappuccino, tea
Pastry, sandwiches	croquette, pastel, rolls, croissant, sandwiches
Butter and margarine	salted and unsalted butter and margarine
Beef	beef
Cookies	simple cakes, sweet breads
Yogurt and dairy products	yogurts, vitamins with milk
Semi-skimmed and skimmed milk	semi-skimmed and skimmed milk
Whole milk	whole milk
Breads, toasts, crackers	various breads, toasts, crackers and whole grains breads
Natural flavorings	garlic, herbs, onion, pepper, oregano, basil, parsley
Chocolate powder	instant breakfast drink
Vegetables	argula, cabbage, chard, escarole, kale, lettuce, spinach, watercress, beets, broccoli, carrots, cauliflower, chayote, cucumber, eggplant, okra, squash, tomato, turnip, mushrooms, carrots, turnip, potato, corn, green peas, olives
Cheeses	cheddar, mozzarella, parmesan, provolone, ricotta, cream cheese, American cheese
Salad dressing	oils, salt, vinegar
Candies	chocolates, gum drops,
White meat	Poultry (chicken, turkey) and Fishes
Processed meats and cold cuts	hamburger, meatballs, sausage, bacon, canned fish, ham, bologna, salami
Sodas	sodas (diet or regular)
Juices	juices or flavored drinks, fresh juice
Pasta	spaghetti, gnocchi, lasagna, ravioli
Sauces	mayonnaise, ketchup, mustard, tomato sauce, soy sauce, white sauce, hot pepper
Eggs	egg boiled, eggs fried, omelet, scrambled egg
Fruits	apple, avocado, banana, grapes, guava, lemon, mango, melon, orange, papaya, peach, pear, persimmon, pineapple, tangerine, watermelon
Ice cream	ice cream and milk shakes
Cakes and pies	cake, pie, pudding, topping
Snacks	chips, manioc chips
Pork meat	pork meat

**Table 2:** Distribution of the total and the components of the Brazilian Healthy Eating Index – Revised (B HEI-R) scores, according to the gender and for the overall population study. São Paulo.

			Percentiles						
Components	Maximum Allowed	Mean ± SD	5%	10%	25%	50%	75%	90%	95%
**Male**									
Total grains	5.00	4.98 ± 0.10	4.95	5.00	5.00	5.00	5.00	5.00	5.00
Whole grains	5.00	0.20 ± 0.29	0.00	0.02	0.04	0.10	0.24	0.49	0.72
Total fruit ^a^	5.00	1.02 ± 0.57	0.15	0.33	0.60	0.97	1.39	1.80	2.02
Whole fruit ^b^	5.00	0.29 ± 0.30	0.01	0.04	0.09	0.20	0.40	0.67	0.90
Total vegetables	5.00	0.85 ± 0.44	0.34	0.40	0.54	0.76	1.06	1.42	1.69
Dark green and Orange vegetables and Legumes ^c^	5.00	4.03 ± 1.24	1.53	2.01	3.10	4.90	5.00	5.00	5.00
Milk ^d^	10.00	4.07 ± 2.45	0.22	1.00	2.29	3.88	5.57	7.28	8.42
Meat, egg and beans ^e^	10.00	8.16 ± 0.94	6.63	6.94	7.51	8.15	8.82	9.46	9.86
Oils ^f^	10.00	9.99 ± 0.11	10	10	10	10	10	10	10
Saturated fat	10.00	6.39 ± 1.56	3.58	4.28	5.36	6.64	7.66	8.27	8.49
Sodium	10.00	1.90 ± 1.09	0.00	0.36	1.11	1.91	2.68	3.33	3.71
SoFAAS ^g^	20.00	4.42 ± 2.77	0.00	0.39	2.34	4.37	6.43	8.12	9.10
Overall BHEI-R	100.00	46.3	27.41	30.77	37.98	46.88	54.25	60.84	64.91
**Female**									
Total grains	5.00	4.98 ± 0.12	4.88	5.00	5.00	5.00	5.00	5.00	5.00
Whole grains	5.00	0.39 ± 0.48	0.02	0.04	0.09	0.22	0.49	0.92	1.34
Total fruit ^a^	5.00	1.50 ± 0.69	0.42	0.62	1.00	1.47	1.98	2.42	2.68
Whole fruit ^b^	5.00	0.50 ± 0.45	0.05	0.09	0.19	0.37	0.68	1.08	1.40
Total vegetables	5.00	1.17 ± 0.61	0.46	0.55	0.74	1.04	1.44	1.96	2.31
Dark green and Orange vegetables and Legumes ^c^	5.00	4.46 ± 0.98	2.15	2.79	4.26	5.00	5.00	5.00	5.00
Milk ^d^	10.00	4.91 ± 2.74	0.78	1.49	2.93	4.68	6.58	8.5	9.79
Meat, egg and beans ^e^	10.00	7.85 ± 1.08	6.08	6.45	7.13	7.85	8.61	9.3	9.72
Oils ^f^	10.00	9.97 ± 0.22	10	10	10	10	10	10	10
Saturated fat	10.00	5.20 ± 1.86	1.9	2.71	3.97	5.28	6.58	7.64	8.11
Sodium	10.00	2.45 ± 1.18	0.41	0.88	1.64	2.48	3.27	3.98	4.38
SoFAAS ^g^	20.00	4.59 ± 2.87	0	0.39	2.43	4.57	6.66	8.38	9.4
Overall BHEI-R	100.00	47.97	27.15	31.01	39.38	47.96	56.29	64.18	69.13
**Overall population**									
Total grains	5.00	4.98 ± 0.11	4.95	5.00	5.00	5.00	5.00	5.00	5.00
Whole grains	5.00	0.29 ± 0.40	0.01	0.02	0.06	0.15	0.35	0.71	1.05
Total fruit ^a^	5.00	1.26 ± 0.68	0.25	0.43	0.75	1.20	1.70	2.18	2.45
Whole fruit ^b^	5.00	0.39 ± 0.39	0.03	0.05	0.12	0.27	0.53	0.90	1.18
Total vegetables	5.00	1.01 ± 0.55	0.37	0.45	0.62	0.89	1.25	1.71	2.07
Dark green and Orange vegetables and Legumes ^c^	5.00	4.24 ± 1.14	1.75	2.30	3.60	5.00	5.00	5.00	5.00
Milk ^d^	10.00	4.48 ± 2.63	0.49	1.22	2.58	4.26	6.08	7.93	9.16
Meat, egg and beans ^e^	10.00	8.01 ± 1.03	6.30	6.69	7.32	8.01	8.72	9.39	9.80
Oils ^f^	10.00	9.98 ± 0.18	10	10	10	10	10	10	10
Saturated fat	10.00	5.80 ± 1.81	2.54	3.36	4.61	5.94	7.20	8.11	8.36
Sodium	10.00	2.18 ± 1.17	0.10	0.57	1.34	2.18	2.99	3.70	4.12
SoFAAS ^g^	20.00	4.51 ± 2.82	0.00	0.39	2.38	4.47	6.55	8.26	9.24
Overall BHEI-R	100.00	47.13	26.79	30.48	38.38	47.37	55.37	62.89	67.43

^a^ Includes 100% juice; ^b^ Includes all forms except juice; ^c^ Includes legumes only after meat and beans standard is met; ^d^ Includes all milk products, such as fluid milk, yogurt and cheese, and soy beverages; ^e^ Includes legumes only if the meat and beans standard is otherwise not met; ^f^ Includes nonhydrogenated vegetable oils and oils in fish, nuts, and seeds; ^g^ SoFAAS–Total calories from solid fats, alcoholic beverages and added sugar.

**Table 3:** Factor loading for dietary patterns obtained in factor analysis (*n* = 229). São Paulo.

	Dietary Patterns	
Food Itens	Traditional	Dual
Beans and Legumes	0.74	−0.07
Rice	0.65	−0.11
Coffee, teas	0.54	−0.26
Sugar	0.53	0.07
Butter and margarine	0.45	−0.27
Beef	0.40	0.10
Cookies	0.35	0.10
Pastry, sandwiches	−0.45	−0.08
Breads, toasts, crackers	−0.31	0.32
Chocolate powder	−0.02	0.66
Vegetables	0.10	0.56
Whole milk	0.24	0.56
Salad dressing	0.84	0.50
Cheeses	−0.25	0.47
Processed meats and Cold cuts	−0.01	0.35
Candies	−0.14	0.31
Juices	0.06	0.30
White meat	0.18	0.28
Fruits	0.10	0.24
Sodas	−0.11	0.20
Semi-skimmed and skimmed milk	−0.08	0.19
Natural flavorings	0.23	0.18
Sauces	0.04	0.12
Eggs	0.14	0.10
Snacks	0.01	0.07
Ice cream	−0.04	0.06
Cakes and pies	−0.167	0.06
Yogurt and dairy products	−0.13	−0.02
Pork meat	0.03	−0.05
Pasta	0.01	−0.09
% Explained Variance	9.02	8.06
Accumulative %	9.02	17.08

Factor loadings ≥|0.30| were considered significant.

**Table 4:** Associations between the dietary patterns and the characteristics of the adolescents living in São Paulo.

		Traditional Pattern				Dual Pattern			
		Linear Regression				Linear Regression			
		Univariate		Multivariate		Univariate		Multivariate	
	*n* (%)	β	*p*	β	*p*	β	*p*	β	*p*
Age (years)	mean (sd): 17.72 (1.18)	0.17	0.01	0.12	0.09	−0.04	0.48	0.09	0.25
Gender									
female	117 (51%)	1.00		1.00		1.00		1.00	
male	112 (49%)	−0.69	*p* < 0.01	−0.70	*p* < 0.01	0.08	0.57	−0.05	0.71
Smoking habits									
non-smoker	199 (87%)	1.00		-		1.00		-	
Smoker/former smoker	30 (13%)	0.37	0.06			−0.16	0.42		
Alcoholic beverages									
no	72 (31%)	1.00		-		1.00		-	
yes	157 (67%)	−0.02	0.88			0.02	0.89		
Family head’s schooling									
low	128 (58%)	1.00		1.00		1.00		1.00	
medium	73 (33%)	−0.30	0.04	−0.23	0.13	0.36	0.01	0.26	0.10
high	21 (9%)	−0.73	0.01	−0.59	0.05	0.83	*p* < 0.01	0.56	0.03
Family head’s marital status									
living alone	67 (29%)	1.00		-		1.00		1.00	
living with a partner/married	162 (71%)	0.13	0.39			0.33	0.02	0.32	0.04
Weight status									
normal weight ¹	134 (68%)	1.00		-		1.00		1.00	
overweight	21 (11%)	0.10	0.65	0.14	0.51	0.17	0.43	−0.03	0.90
obesity	42 (21%)	0.22	0.19	0.09	0.60	−0.18	0.28	0.01	0.99
Adolecent’s remunerated activity									
no	119 (53%)	1.00		-		1.00		-	
yes	105 (47%)	0.13	0.32			0.01	0.96		
Family income ²									
<1 minimum wage	70 (35%)	1.00		1.00		1.00		1.00	
≥1 minimum wage	129 (65%)	−0.45	0.01	−0.31	0.04	0.50	0.01	0.48	0.01

^1^ Only two adolescents were classified as underweight; ^2^ Minimum wage at the period of collection was US $75.00.
